# MYNursingHome: A fully-labelled image dataset for indoor object classification.

**DOI:** 10.1016/j.dib.2020.106268

**Published:** 2020-09-03

**Authors:** Asmida Ismail, Siti Anom Ahmad, Azura Che Soh, Mohd Khair Hassan, Hazreen Haizi Harith

**Affiliations:** a Department of Electrical and Electronic Engineering, Faculty of Engineering, Universiti Putra Malaysia, Serdang 43400, Malaysia; b Department of Biological and Agricultural Engineering, Faculty of Engineering, Universiti Putra Malaysia, Serdang 43400, Malaysia; c Department of Engineering & Technology, Faculty of Technical & Vocational, Universiti Pendidikan Sultan Idris, Tanjung Malim 35900, Malaysia; d Malaysian Research Institute on Ageing (MyAgeing™), Universiti Putra Malaysia, Serdang 43400, Malaysia

**Keywords:** Image dataset, Indoor objects, Deep learning, Object detection, Object classification

## Abstract

A fully labelled image dataset serves as a valuable tool for reproducible research inquiries and data processing in various computational areas, such as machine learning, computer vision, artificial intelligence and deep learning. Today's research on ageing is intended to increase awareness on research results and their applications to assist public and private sectors in selecting the right equipments for the elderlies. Many researches related to development of support devices and care equipment had been done to improve the elderly's quality of life. Indoor object detection and classification for autonomous systems require large annotated indoor images for training and testing of smart computer vision applications. This dataset entitled MYNursingHome is an image dataset for commonly used objects surrounding the elderlies in their home cares. Researchers may use this data to build up a recognition aid for the elderlies. This dataset was collected from several nursing homes in Malaysia comprises 37,500 digital images from 25 different indoor object categories including basket bin, bed, bench, cabinet and others.

## Specifications Table

**Table:** 

Subject	Electrical and Electronic Engineering
Specific subject area	Image processing, Image identification, Image classification, object detection, computer vision, artificial intelligence, deep learning
Type of data	Image
How data were acquired	Images captured using a single camera.
Data format	JPG, Raw
Parameters for data collection	Original data were collected by capturing videos of indoor objects commonly used by elderlies.
Description of data collection	The data is manually collected with the help of elderly home care management staff. Images are manually cropped to remove any background or surrounding objects during the learning process.
Data source location	Darul Hanan, Lot 2965, Mukim 6, Pongsu Seribu 13200 Kepala Batas, Pulau Pinang. Bait Al-Mawaddah, Lot 140452 Jalan Tanjung Pahang, Lorong Haji Mughani, Jalan Kebun Tambahan, Seksyen 30, 40460 Shah Alam, Selangor.
Data accessibility	Dataset can be accessible at Mendeley data: http://dx.doi.org/10.17632/fpctx3svzd.1

## Value of the Data

• MYNursingHome dataset can be used to develop indoor object detection system and navigation assist device for the elderlies. Current indoor datasets mainly focus on scenes and common objects in workplace or house. MYNursingHome dataset focus is on objects in elderly living institutions’ surrounding.
• MYNursingHome dataset could be a useful resource for researchers in the fields of computer vision, deep learning and advanced image classification community. It can be used to test and compare different computer visions and image processing classifiers in detecting different objects based on their visual features. The proposed dataset is original and can be adopted in designing the perception system for an autonomous robot, humanoid robot or mobility assistive devices. These can facilitate researchers to develop support devices for elderlies with vision constraint and disabilities in healthcare.
• MYNursingHome dataset presented in this paper is a ready-to-use database that can directly be used in the field of computer vision to develop new algorithms that can be easily integrated with many other applications.
• MYNursingHome dataset will be used in the autonomous platform perception system inside the elderlies home care in Malaysia.  The objects presented in this dataset are primarily focused for the elderly use but at the same time it can also benefit general public especially the disabled and visually impaired personnel.

## 1 Data Description

MyNursingHome is a fully labelled image dataset collected from several elderly home care centers in Malaysia to supplement the widespread use of image for a variety of computer processing areas such as classification, recognition, segmentation and detection. In general, the elderly suffers from various problems and disorders such as, memory difficulty, short sightedness and dementia. Most of them are unable to manage their daily routine on their own because of these problems. Intelligent assistive system for indoor object detection and recognition is required to encourage these people to move independently.

There are some related works on elderly object recognition assist system that used standard benchmark dataset to identify objects. In [1] the authors present an indoor object recognition system to classify different indoor objects in order to improve the life quality of the elderlies. The datasets used for these experiments consist of 347 images of eight different indoor objects. In [2], the authors proposed scene recognition and indoor object detection system using MIT 67 indoor dataset and 15 scene datasets. Authors in [3] proposed a rough understanding of surrounding objects for people with visual impairment, including the elderly, applied to indoor spaces. The dataset was manually built at two different indoor spaces with 130 images for every 15 object classes. However, those small datasets can affect the robustness and efficiency of the system development.

**Fig. 1:**
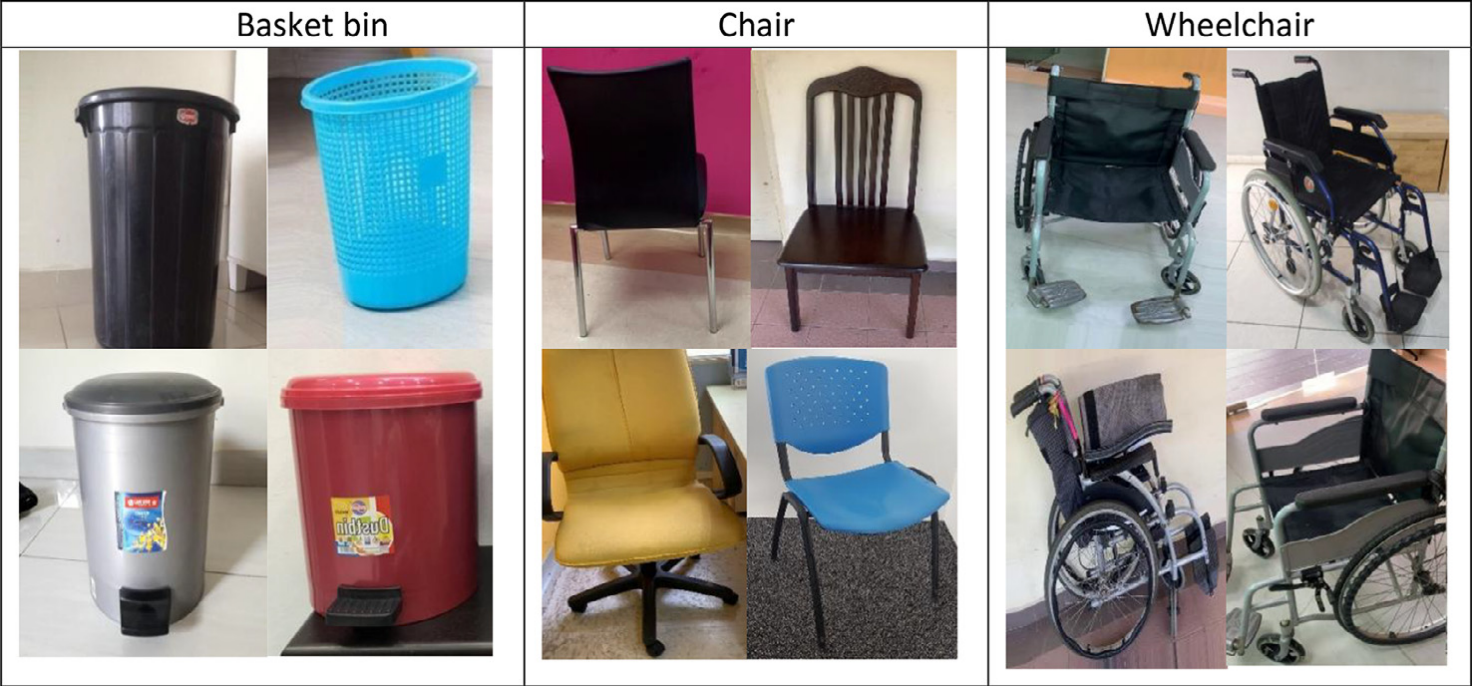
Some of the images and categories in MYNursingHome dataset.

**Fig. 2:**
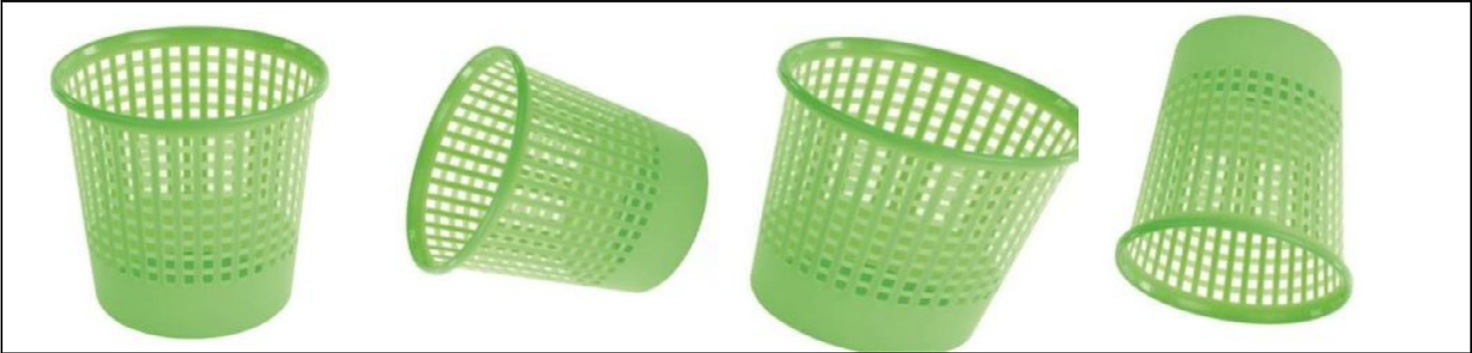
Example of an object in MYNursingHome dataset at different orientations.

MYNursingHome is very practical as the dataset contains 37,500 digital images from 25 different indoor object categories commonly found in the elderly's home care centers, including basket bin, bed, bench, cabinet, call bell, cane stick, chair, door, electric socket, fan, fire extinguisher, handrail, human being, rack, refrigerator, shower, sink, sofa, table, television, toilet seat, walker, wardrobe, water dispenser and wheelchair. Fig. 1 presents sample images from the object categories covered by MYNursingHome dataset. These various objects’ pictures were captured in various positions and angles.  To cater for numerous data variations, the original images were randomly rotated and modified using augmentation process through simple geometric transformation. Fig. 2 shows one of the objects in the dataset at different orientations after going through augmentation process. There are many potential applications of this dataset in the development of combined computer vision and machine learning algorithms to assist people with visual impairment during mobility, especially in unfamiliar surroundings such as clinics, hospitals and other public areas.

## 2 Experimental Design, Materials, and Methods

### 2.1 Camera specification

The dataset is gathered using iPhone XS Max main camera.  The dual rear camera lens has 12-megapixel, wide-angle sensor with an f/1.8 aperture and f/2.4 in telephoto, optical image stabilization and 1.4 nm pixel size. These pixels are much larger and deeper, allowing for more light into the sensor and both rear sensors has OIS feature. Videos captured by this camera has 2160p@24/30/60fps, 1080p@30/60/120/240fps, HDR, stereo sound recording. RGB colour range is chosen for each of these images in the JPG combination.

### 2.2 Building dataset

**Fig. 3:**
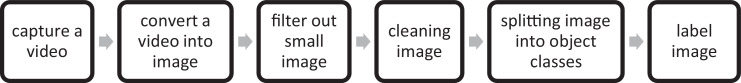
Process flow diagram in developing a dataset.

Fig. 3 shows the processes involved in developing a dataset. The videos captured during data collection were processed in real time. After which, the recorded video will be converted into an image using a program that saves frames from a video file to JPG image series. In this conversion process, the image was captured from the video at 3 s/frame. There are some processes that needed to be carried out before the data can be analysed. The first process is filtering out small images. This process is needed to ensure that the images have certain threshold that helps omit super low-quality images. After filtering small images, the next process is data cleaning that filters the data by removing unwanted images for it to be easier to explore, understand and model the images. After cleaning process, the images need to be split into group/classes according to their categories. MYNursingHome consists of 25 classes with 1500 images per class where there are duplicate image from the same class randomly modified using augmentation process. The images were labelled and connected together where images should be called in order or sequence according to its class. For each class, the number of images should approximately be standardized as per the number of images per class.

## Declaration of Competing Interest

None.

## Appendix Supplementary materials

Image, application 1
